# Evaluation of the efficacy of supplementary probiotic capsules with topical clobetasol propionate 0.05% versus topical clobetasol propionate 0.05% in the treatment of oral lichen planus (a randomized clinical trial)

**DOI:** 10.1186/s12903-024-05246-x

**Published:** 2025-03-05

**Authors:** Yasmine Kamal, Amira Abdelwhab, Sherifa Tarek Salem, Mariam Fakhr

**Affiliations:** 1 Department of Oral Medicine and Periodontology, Faculty of Dentistry, Cairo University, Cairo, Egypt; 2 Department of Oral Medicine and Periodontology, Faculty of Dentistry, October 6 University, Cairo, Egypt; 3 Department of Clinical and Chemical Pathology, Faculty of Medicine, Cairo University, Cairo, Egypt

**Keywords:** Oral lichen planus, OLP, Probiotics, Clobetasol Propionate

## Abstract

**Objectives:**

Probiotics are live microorganisms consisting of many bacterial species that have immunoregulatory functions. The effectiveness of probiotic administration in conjunction with topical corticosteroid application in oral lichen planus (OLP) treatment was evaluated.

**Methods:**

Sixty OLP patients were enrolled in this study and divided into two groups. Group 1 (Probiotics’ group): probiotic capsules were administered twice daily, for 4 weeks in addition to topical clobetasol propionate application 0.05% four times daily. Group 2 (Control group): topical clobetasol propionate 0.05% was applied 4 times daily for 4 weeks. Thongprasom criteria, numerical rating scale and candidal load were evaluated.

**Results:**

Significant reduction in the numerical rating scale as well as Thongprasom scale in the probiotic group when compared to the control group, after 2 and 4 weeks, and more significant reduction was observed after 2 weeks. No difference in the reduction of the candidal load was observed between the two groups, nevertheless, no topical antifungal was used in the intervention group.

**Conclusions:**

Systemic administration of probiotics as a supplementary treatment with topical corticosteroid was effective regarding the reduction of; pain, Thongprasom scales, and candidal load. However, the effectiveness was more evident after 2 weeks when compared to 4 weeks resulting in: more rapid relief of symptoms, improving quality of life, in addition to their antifungal properties.

**Trial registration:**

The current study was registered in *clinicaltrials.gov (ID: NCT04383236) 6-11-2023*.

## Background

Oral lichen planus (OLP) is a multifactorial immunologic mucocutaneous disorder with a global incidence of about 1%, and a 1.5:1 female to male sex predilection [1]. In Egypt, the incidence of OLP is 1.43%, with a female to male ratio of about 2.2:1 [2]. Although the etiology of OLP is idiopathic, however, several risk factors may be associated. Genetic predisposition, psychological stress, and viral infections are considered the main risk factors for OLP development [3].

OLP manifests itself either as oral lesions only, or as intraoral and extraoral lesions. It is manifested as white plaque, reticular-like, papular, atrophic, or bullous erosive bilateral lesions. Orally: buccal mucosae, tongue, gingivae and labial mucosae are commonly affected [4].

Several mechanisms have been implicated in the pathogenesis of lichen planus. Antigen specific mechanism involves antigen presentation by basal epithelial cells, with subsequent killing by CD8 T-cells. Nonspecific mechanisms include; mast cell degranulation and matrix metalloproteinases activation, with subsequent tissue damage [5].

Clobetasol propionate (CP) is one of the superpotent forms of topical corticosteroids and it has anti-inflammatory effect through prevention of inflammatory processes such as edema, fibrin deposition, vasodilation, and phagocytic activity [6]. However, numerous adverse effects of corticosteroid application are documented including oral candidiasis with associated burning sensation, hypogeusia, and adrenal atrophy [7]. In addition, topical clobetasol gel may result in adrenal suppression especially if used for long duration [8].

Host-modulating bacteria application is one of the promising therapies. Probiotics bacteria are live microorganisms that consist of many species such as; *Lactobacillus*,* and Bifidobacterium*. When administered in adequate amounts, they can exert a beneficial effect on the host’s immune response [9]. Their immunoregulatory functions are quite different as they can regulate the immune response in a strain-specific manner. Probiotics are able to modulate the immune response towards the anti-inflammatory cytokines production. The impact of probiotic administration is demonstrated to be beneficial without worsening pre-existent conditions [10]. Moreover, Probiotics can hinder the candidal growth through the production of antimicrobial products as, organic acids (lactic acid, acetic acid), bacteriocins, and hydrogen peroxide, which all act to reduce the growth of candidal species [11].

It has also been reported that probiotics have useful effects on some immunologic disorders, including rheumatoid arthritis, inflammatory bowel disease, and atopic dermatitis [12]. Probiotics can be used as a beneficial treatment option for OLP because they have several immunomodulatory and anti-inflammatory effects [13].

Based on the mechanism of probiotics in modulating the local and systemic inflammatory immune processes, it is feasible to investigate their effects on OLP. Therefore, this study aimed to explore the effectiveness of probiotic administration, in conjunction with topical corticosteroid application in the treatment of atrophic and erosive OLP; as well as, evaluating their antifungal properties.

## Methods

The study is a randomized, single-blinded controlled clinical trial. Sixty patients were recruited among patients who are clinically diagnosed as OLP from the Department of Oral Medicine and Periodontology, Faculty of Dentistry, Cairo University; and October 6 University, between January 2023 to December 2023. They were equally divided to be either in group **P** (probiotics group), or group **C** (control group). Sample size was performed using G*Power version 3.1.99.7. The study’s protocol and consent form were approved by the research ethics committee, Faculty of Dentistry, Cairo University (311122); and were registered in *clinicaltrials.gov (ID: NCT04383236).* The current study adheres to CONSORT guidelines. A thorough clinical examination was performed for all participants, followed by an incisional biopsy for histopathologic evaluation to confirm diagnosis. After the explanation of all aspects of the study, a signed informed consent form was obtained from all participants.

### Inclusion criteria

Male or female patients between 20 and 75 years old, who were diagnosed with symptomatic OLP whether bullous erosive or atrophic subtypes were included. The patients were selected, if they had symptomatic painful intra-oral lesions associated to OLP at the time of recruitment, with minimum severity of pain being ≥ 3 on a 0–10 numeric pain rating scale.

### Exclusion criteria

The use of systemic antibiotics, corticosteroids, or immunosuppressive medications within four weeks before enrollment in the study. Pregnant females, patients with a history of any systemic disorder affecting the immune system, malignancies, and hepatic patients were also excluded. In addition to allergy, or intolerance to probiotics.

#### Randomization and allocation

Enrolled patients were randomly distributed between the two groups using an online randomization program: http://www.randome.org. To achieve allocation concealment, randomization was performed by an examiner who was not involved in the study.

***The intervention group (Group P)*** was randomly allocated to take one capsule of probiotics complex obtained from *Biovea*, twice daily; during mealtime at the morning and evening, for the 4 weeks study period. Moreover, patients in this group were instructed to apply clobetasol propionate 0.05% in orabase gel 4 times daily for 4 weeks; 3 times after meals and once before sleeping for 4 weeks. Patients had been receiving their supply of the gel and probiotics every 2 weeks. Patients were informed to contact us if any side effects were developed during, or after treatment.

The probiotics complex is composed of: probiotics mixture of 4 probiotic strains 200 mg: *Lactobacillus acidophilus 3.2 billion CFU*,* Lactobacillus bulgaricus 40 million CFU*,* Bifidobacterium bifidum 200 million CFU*,* Streptococcus thermophilus 200 million* CFU. These probiotics’ strains are combined reaching (4 × 10^9^ CFU) per capsule.

***The control group (Group C)*** was randomly allocated to apply clobetasol propionate 0.05% in orabase gel 4 times daily; 3 times after meals and once before sleeping, for 4 weeks. At the 3rd week, this group was instructed to apply miconazole gel 4 times daily as an anti-fungal prophylaxis for two weeks.

### For salivary sample collection

Salivary samples were collected from all participants early in the morning at 8 am. Participants were instructed to avoid food high in sugar content, caffeine, or acidity because it may result in lowering salivary pH which may compromise the assay. Individuals were instructed to rinse their mouths and wait for 10 min to avoid sample dilution before collecting saliva [14]. Five milliliters of whole unstimulated salivary samples were collected by spitting into a graduated clear test tube, to be used in the evaluation of candidal counts before and after treatment.

Patient’s visits were scheduled as follow: at baseline, 2 weeks, and 4 weeks. All patients were regularly reminded about their coming visit by a phone call. At the follow up visits, patients were instructed to bring back the consumed container, to check that they had totally consumed their previous supply before giving them the new refill.

Intraoral examination was performed using visual and tactile examination techniques to examine the oral mucosa. Size of the lesion of OLP was measured and assessed, as well as the patient’s pain score. Any signs of erythematous or pseudomembranous candidal infection were checked and examined as well. All together with, intraoral photographs preoperatively and postoperatively were taken, to compare the clinical improvement before and after the treatment in both groups.

**Primary outcomes** were 1- *The size of the lesion*: which was recorded at baseline, 2 weeks, and after 4 weeks of the study; and it was scored by Thongprasom scale which is categorized as follows: 0 = no lesion, 1 = white streaks with no erythema, 2 = white streaks with atrophic areas < 1 cm², 3 = white streaks with atrophic areas > 1 cm², 4 = white streaks with erosive areas < 1 cm², 5 = white streaks with erosive area > 1 cm² or ulceration **(Thongprasom et al.**,** 1992).** 2) *Pain*: was recorded 3 times; at the beginning, after 2 weeks and at the end of the 4 weeks study period. Pain was graded by numerical rating scale (NRS); which is composed of a 10-cm horizontal line between extremities, with (0) indicating no pain, and (10) for unbearable pain.

**Secondary outcome** was *Candidal load assessment*: Candidal load was measured through culturing technique in CFU (Colony forming unit) for the two groups, at baseline and at the end of the 4-week study period; to detect the effect of the prophylactic probiotics consumption on the reduction of the candidal overload. The salivary samples were plated in Sabouraud dextrose agar supplemented with gentamicin (20 mg/mL) and was incubated in the incubator at 37 °C for 48 h. The number of colony forming units *per* mL of saliva (CFU/mL) was counted.

### Statistical analysis

The Categorical data were presented as frequency and percentage values and were analyzed using Fisher’s exact test. Numerical data was represented as mean, standard deviation (SD), median, and interquartile range (IQR) values. They were analyzed for normality by checking data distribution and using Shapiro-Wilk’s test. Age data were normally distributed and were analyzed using independent t-test. Other non-parametric data were analyzed using the Mann-Whitney U test for intergroup comparisons and Friedman’s test, followed by the Nemenyi post hoc test for intragroup comparisons. *P*-values were adjusted for multiple comparisons using the False Discovery Rate (FDR) method. Correlations were analyzed using Spearman’s rank-order correlation coefficient. The significance level was set at *p* < 0.05 within all tests. Statistical analysis was performed with R statistical analysis software version 4.3.2 for Windows (R Core Team (2023). R: A language and environment for statistical computing. R Foundation for Statistical Computing, Vienna, Austria. URL https://www.R-project.org/.*).* The present study was conducted following consort guideline.

## Results

Sixty OLP patients were randomly and equally allocated to the intervention group (group P) and the control group (Group C). In the probiotic group, there were 11 males and 19 females with a mean age of (48.47 ± 11.50) years, while in the control group, there were 9 males and 21 females with a mean age of (46.77 ± 10.10) years. There was no significant difference between both groups regarding different demographic data (*p* > 0.05) Table 1. Probiotic administration was well tolerated by all patients. No patient reported any adverse effect from probiotic administration. All patients enrolled in the study completed the trial.

**Table 1:** Summary statistics and intergroup comparisons of demographic data

Parameter		Probiotic	Control	Test statistic	*P*-value
Gender [n (%)]	***Male***	11 (36.67%)	9 (30.00%)	**0.30**	**0.785**
	Female	19 (63.33%)	21 (70.00%)		
Age (years)	Mean ± SD	48.47 ± 11.50	46.77 ± 10.10	**0.61**	**0.545**
	Median (IQR)	48.00 (14.00)	46.00 (16.50)		

At baseline, there was no significant difference between NRS values in the probiotic 8.00 (4.00) and the control groups 7.50 (4.00) (*p* = 0.313). After 2 and 4 weeks, a significant reduction in the NRS in the probiotic group 2.00 (4.00)^B^ & 0.00 (3.00)^C^ was detected, when compared with the control group 5.00 (4.00)^B^ & 2.50 (1.75)^C^ (*p* < 0.05). Within both groups, there was a significant reduction in measured scale with time (*p* < 0.001) (Table 1).

**Fig. 1:**
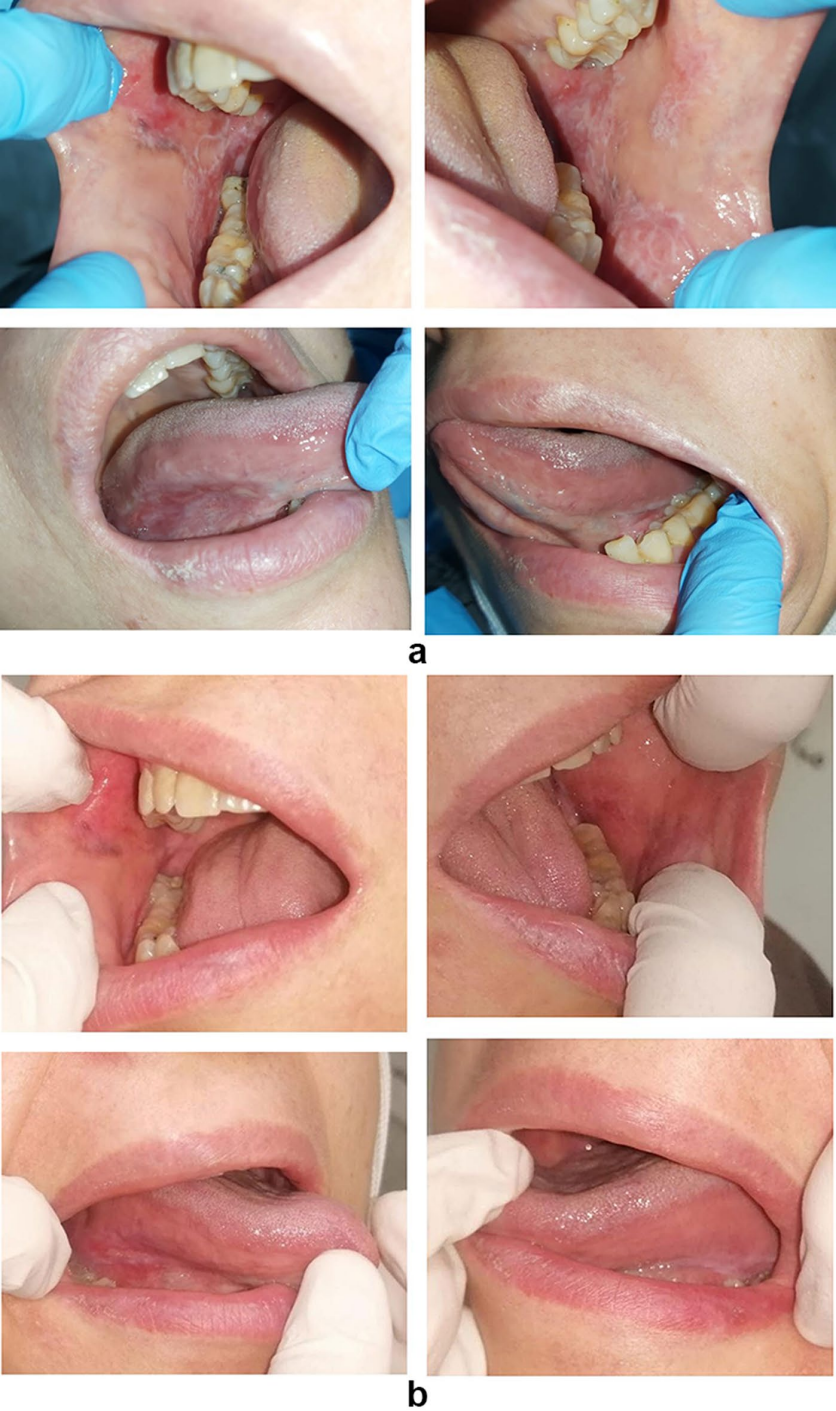
**a**. Clinical photograph for a fifty one years old female patient before treatment (group P). **b**. The same patient after treatment (group P)

Regarding Thongprasom scale, there was no significant difference between both groups at baseline (*p* = 0.738). After 2 and 4 weeks, there was significant reduction in the probiotic group 3.50 (13.00)^B^ & 3.00 (4.00)^C^, when compared to the control group 7.50 (7.00)^B &^ 3.00 (15.00)^C^ (*p* < 0.05). Within both groups, the measured scale was significantly reduced with time (*p* < 0.001) (Table 2, Fig. 1).

**Table 2:** Summary statistics, inter and intragroup comparisons of NRS and the Thongprasom scale

		NRS				Thongprasom scale			
Interval	Measurement	Probiotic	Control	Test statistic	*p*-value	Probiotic	Control	Test statistic	*p*-value
Baseline	Mean ± SD	7.70 ± 1.93^A^	7.13 ± 2.18^A^	517.50	0.313	21.40 ± 14.53^A^	21.08 ± 19.09^A^	473.00	0.738
	Median (IQR)	8.00 (4.00)^A^	7.50 (4.00)^A^			14.25 (29.50)^A^	11.50 (13.50)^A^		
2 weeks	Mean ± SD	2.33 ± 1.97^B^	4.97 ± 2.09^B^	727.50	< 0.001*	9.00 ± 10.21^B^	14.20 ± 13.80^B^	657.00	0.002*
	Median (IQR)	2.00 (4.00)^B^	5.00 (4.00)^B^			3.50 (13.00)^B^	7.50 (7.00)^B^		
4 weeks	*Mean ± SD*	1.20 ± 1.56^C^	2.83 ± 1.97^C^	660.00	0.001*	6.35 ± 8.43^C^	10.02 ± 11.79^C^	612.00	0.012*
	Median (IQR)	0.00 (3.00)^C^	2.50 (1.75)^C^			3.00 (4.00)^C^	3.00 (15.00)^C^		
Test statistic		56.59	56.00			55.71	52.30		
p-value		< 0.001*	< 0.001*			< 0.001*	< 0.001*		

SD Standard deviation, IQR Interquartile range, values with *different superscripts* within the *same vertical column* are significantly different, *Significant (*p* < 0.05)

At both intervals, the percentage reduction in both scales from baseline was significantly higher in the probiotic group, than in the control group (*p* < 0.001). Regarding NRS, the percentage of reduction was significantly higher in the probiotic group, 69.05 (40.00) & 100.00 (30.00); when compared to the control group, 35.42 (11.43) & 60.00 (4.64), after 2 & 4 weeks respectively. Similarly, in the Thongprasom scale, the percentage of reduction was significantly higher in the probiotic groups, 72.89 (17.08) & 80.62 (11.27), when compared to the control group 30.77 (6.91) & 64.58 (25.64), after 2 & 4 weeks respectively (Table 3).

**Table 3:** Summary statistics and intergroup comparisons of percentage of reduction in measured scores

Scale	Interval	Measurement	Probiotic	Control	Test statistic	*p*-value
NRS	Baseline- 2 weeks	Mean ± SD	72.89 ± 21.57	32.68 ± 12.11	**855.00**	< 0.001*
		Median (IQR)	69.05 (40.00)	35.42 (11.43)		
	Baseline- 4 weeks	Mean ± SD	87.00 ± 17.65	60.69 ± 21.21	**748.50**	< 0.001*
		Median (IQR)	100.00 (30.00)	60.00 (4.64)		
Thongprasom scale	Baseline- 2 weeks	Mean ± SD	66.76 ± 19.21	33.97 ± 10.17	**789.00**	< 0.001*
		Median (IQR)	72.89 (17.08)	30.77 (6.91)		
	Baseline- 4 weeks	Mean ± SD	74.36 ± 18.38	56.72 ± 22.25	**748.50**	< 0.001*
		Median (IQR)	80.62 (11.27)	64.58 (25.64)		

SD Standard deviation, IQR Interquartile range, *Significant (*p* < 0.05)

There was no significant difference between both groups regarding the reduction in the candidal count (*p* = 0.819) (Table 4).

**Table 4:** Summary statistics and intergroup comparisons of percentage of reduction in candidal count

Measurement	Probiotic	Control	Test statistic	*p*-value
Mean ± SD (CFU)	29.97 ± 46.56	36.61 ± 48.93	**436.50**	**0.819**
Median (IQR) (CFU)	0.00 (99.75)	0.00 (99.80)		

SD Standard deviation, IQR Interquartile range

There was a strong positive correlation between NRS and Thongprasom scale, and it was statistically significant (rs = 0.808, *p* < 0.001) (correlation coefficient 95%CI).

## Discussion

Probiotics have been used in a range of gastrointestinal tract conditions including, antibiotic-associated diarrhea, and ulcerative colitis in order to enhance the intestinal microbial microflora balance, and to reduce systemic and local inflammation [15]. Additionally, Probiotic administration was effective in recurrent aphthous ulcerations, Behcet’s disease, oral candidiasis, and oral mucositis resulting in the reduction of the size and the number of the presented lesions [16–18].

Thus, the aim of this study, was to evaluate the efficacy of supplementary probiotic capsules in conjunction with topical clobetasol propionate on OLP treatment. To the best of our knowledge, the present study is the first one conducted using the systemic administration of the probiotic mixture containing L. acidophilus, L.bulgaricus, S. thermophilus, and B.bifidum for symptomatic OLP treatment.

Results of the present study showed a significant reduction in the NRS of the probiotic group when compared to the control group, after 2 and 4 weeks. Moreover, a more significant reduction was observed after 2 weeks, which proves their synergistic effect with corticosteroid administration, resulting in more rapid relief of pain with a median range of NRS 2 after 2 weeks.

Probiotics exert a strong anti-inflammatory action through the production of IL-10 or IL-4 as well as decreasing the production of proinflammatory cytokines, such as Tumor necrosis factor (TNF). Furthermore, probiotics are able to modify pain signaling by producing gamma aminobutyric acid (GABA); the most important inhibitory neurotransmitter. In addition Probiotics have antinociceptive effects and can induce a sustained increase in opioid receptor (OPRM1) mRNA expression and also induces significant cannabinoid receptor (CNR2) mRNA expression [19].

However, in Thongprasom scale there was a significant reduction after 2 weeks in most of the cases and the median range was 3.5 after 2 weeks; but less significant between 2 weeks and 4 weeks. This could be attributed to the presence of multiple papular lesions with absence of pain. Probiotics can modulate immune response in a strain-specific, and time/dose-dependent manner [10]. In vitro and animal studies have suggested that probiotics administration can reduce the production of pro-inflammatory cytokines [20, 21]. In addition, they modulate the inflammatory cytokines production, MMP-9 expression, NF-kB signaling pathways, keratinocytes apoptosis, mast cell degranulation and T cell activation [10]. Wang et al., 2020 [22] documented that dysbiosis in the oral microbiome may have a role in OLP treatment and decreasing disease activity. They can stimulate the activity of T regulatory cells, and alleviate several problems in a strain-specific manner [13]. In addition, probiotic administration directs the mucosal response towards the anti-inflammatory cytokines production resulting in reduction of the mucosal damage. Therefore, probiotics could be considered a safe and sustainable immunomodulatory approach for OLP management [10].

At both intervals, the percentage reduction in both scales from baseline was significantly higher in the probiotic group (*p* < 0.001). Moreover, there was a strong positive correlation between both scales that was statistically significant. Regarding the candidal load, there was no significant difference between both groups regarding the reduction in candidal count (*p* = 0.819). Nevertheless, no topical antifungal was used throughout the 4 weeks of the study in the intervention group, owing to the fact that probiotics can be also used as a prophylactic agent against oral candidiasis [23]; while in the control group, a topical antifungal was introduced as a prophylaxis by the 3rd week.

Probiotics were effective in improving the quality of life for all participants because their administration resulted in a more rapid reduction of pain, as well as ulcer size resulting in; better mastication, swallowing, and speaking that were more obvious after 2 weeks when compared with topical corticosteroid.

Our results were in contrast with the study conducted by Marlina et al., 2022 [24] as they had conducted a pilot trial of probiotic sachet supplement (VSL#3) versus placebo. Their study explored the systemic effect of probiotics for OLP treatment. However, they detected that there was no statistically significant change in pain, disease activity, or oral microbial composition when compared to placebo. The difference in results between that study and the current one, could be attributed to the different strains of probiotics used in the two studies as it has been known that probiotics interact in a strain-specific manner.

On the same line to our results, Aggour et al., 2021 [16] studied the effect of probiotics in the treatment of recurrent aphthous stomatitis in adults and children, and they deducted that topical application of probiotics in the form of lozenges could decrease the pain intensity, as well as the acceleration of the healing especially in children. Similarly, Nirmala et al., 2019 concluded that probiotic Bacillus clausii could be considered as a new strategy for treating recurrent aphthous stomatitis as it could reduce the ulcer severity [25].

Furthermore, Kamal et al., 2020 [23] conducted a study investigating the efficacy of multispecies probiotic product containing a mixture of L.bulgaricus, L. acidophilus, S. thermophilus and B.bifidum, and they recommended its use as an effective prophylactic agent for oral candidiasis.

Based on the results of the current study and the previous related studies, probiotics can be highly recommended as promising therapeutic, as well as prophylactic agents to various oral lesions owing to their different immunomodulatory effects.

The limitations of the present study were small sized sample, and lack of a follow up period after completion of treatment to investigate their effect related to decreasing execration periods.

## Conclusion

The results obtained in the present study revealed the clinical efficacy of probiotics, which possess unique characteristics, addressing the requirements of efficacy, tolerability, and accessibility that could make it a valid therapeutic line for OLP treatment. Probiotic supplementation with topical corticosteroid administration results in more rapid relief of symptoms improving quality of life in addition to their antifungal properties.

### Recommendations

Studies to find the effect of probiotics in conjunction with lower doses of corticosteroids are required. In addition, studies with longer follow up periods, and larger sample sizes are recommended to investigate their possible role in decreasing the exacerbation outbreaks.

## Data Availability

The datasets generated during and/or analyzed during the current study are available from the corresponding author on reasonable request.

## Abbreviations

OLP: Oral Lichen Planus
CP: Clobetasol Propionate
NRS: Numerical Rating Scale
